# Comment on “Effectiveness of Naltrexone in the Prevention of Delayed Respiratory Arrest in Opioid-Naive Methadone-Intoxicated Patients”

**DOI:** 10.1155/2014/705754

**Published:** 2014-03-11

**Authors:** Benjamin D. Kessler, Robert S. Hoffman

**Affiliations:** ^1^Department of Emergency Medicine, North Shore University Hospital, Manhasset, NY 11030, USA; ^2^Division of Medical Toxicology, Department of Emergency Medicine, NYU School of Medicine, New York, NY 10016 USA Division of Medical Toxicology, Department of Emergency Medicine, NYU School of Medicine, New York, NY 10016, USA

In the recent paper by Aghabikoolei et al. [1], the authors postulate that oral naltrexone prevents delayed respiratory depression following naloxone reversal of methadone toxicity. We would like to voice a few concerns regarding the study.

The restriction of the study to only opiate naïve patients limits its applicability. Methadone is more frequently used for the prevention of opioid withdrawal symptoms, for the maintenance of opioid abstinence, or for the treatment of chronic pain. Inherently, by the nature of access to the medication, most people who are exposed are tolerant. It seems problematic to include patients based on self- or familial account that they do not use opiates. The number of patients originally enrolled but subsequently excluded for withdrawal symptoms after naloxone administration highlights the challenges of this approach.

Additionally, the half-life of methadone is approximately 48 hours and the 50 mg of naltrexone is expected to block the effects of the methadone at the opioid receptors for approximately 24 hours. Since the patients in the naltrexone group only had a mean hospital stay of 26 hours, many of them left when they were still potentially vulnerable to delayed respiratory depression. It would be important to know if any returned for readmission or were subsequently found dead.

Abrupt and prolonged iatrogenic opioid withdrawal can be severe and life threatening [2] and should be avoided. This must be balanced by the need for ICU level care when respiratory depression is predicted. As such, the results of this study seem most applicable in unintentional ingestions in children since they are usually opiate naïve and often require transfer for PICU level care.

## References

[1] Aghabiklooei A, Hassanian-Moghaddam H, Zamani N (2013). Effectiveness of naltrexone in the prevention of delayed respiratory arrest in opioid-naïve methadone-intoxicated patients. *BioMed Research International*.

[2] Hamilton RJ, Olmedo RE, Shah S (2002). Complications of ultrarapid opioid detoxification with subcutaneous naltrexone pellets. *Academic Emergency Medicine*.

